# The effect of the strain rate on the longitudinal modulus of cellulosic fibres

**DOI:** 10.1007/s10853-022-07722-7

**Published:** 2022-09-22

**Authors:** Marko Zizek, Caterina Czibula, Ulrich Hirn

**Affiliations:** 1 Institute of Bioproducts and Paper Technology, Graz University of Technology, Inffeldgasse 23, 8010 Graz, Austria; 2 CD Laboratory for Fiber Swelling and Paper Performance, Graz University of Technology, Inffeldgasse 23, 8010 Graz, Austria

## Abstract

The longitudinal stiffness of cellulosic fibres plays an important role in the mechanical performance of many products these fibres are used for. Especially, the fibres’ viscoelastic properties are having an influence on the product performance.

In this work, tensile testing at different strain rates was performed on single fibres to investigate the rate dependence of their moduli. Four different fibre types were tested: chemi-thermomechanical softwood pulp (CTMP), bleached chemical softwood pulp (CP), unbleached softwood kraft pulp (UKP), and viscose (VIS). For each fibre sample, ten strain rates ranging from 0.113% s^−1^ to 800% s^−1^ were applied. The rate-dependent modulus $E_{r}$ of each fibre at each strain rate was calculated by linearly fitting the stress–strain curves. By obtaining the values of the normalized modulus, a slope value per decade was calculated to quantify the rate dependence. To exclude possible plastic and relaxation effects, two additional experiments were used: a force-controlled loading–unloading experiment and a reverse rate protocol.

All cellulosic fibres tested exhibited rate-dependent behaviour with a log-linear relationship between loading rate and modulus. For each tenfold increase in loading rate we found an increase in modulus up to nearly 20%—depending on the fibre type. Viscose fibres exhibit the highest rate dependence, whereas chemical softwood pulp fibres exhibit the lowest.

**Supplementary Information:**

The online version contains supplementary material available at 10.1007/s10853-022-07722-7.

## Introduction

In the development of fibre-based products and processes, single fibre testing is employed to improve the understanding of the mechanical relationship between single fibres and the final fibre-based product. An example of such a product is paper, which consists of a network of wood pulp fibres. The most established technique to characterize single pulp fibres is tensile testing and over the years, several structural- and process-dependent effects have been investigated [1–7]. Pulp fibre tensile stiffness was found to be affected by the microfibril orientation [5] and moisture content [6]. However, determining the effects of different chemical and mechanical treatment in commercial pulps is not straightforward since the fibre properties exhibit a high variation from sample to sample [7].

Wood fibres consist mainly of cellulose, hemicellulose, and lignin assembled into a structure with several wall layers [8, 9]. The fibre wall layers differ in chemical composition, cellulose microfibril alignment, and layer thickness. Whereas in the primary layer the lignin content is rather high and the cellulose microfibrils are randomly aligned, the secondary wall exhibits a higher degree of fibril alignment and is further divided into three layers: S1, S2, and S3. The S2 layer is the thickest layer and the microfibrils tend to be highly oriented, therefore, the S2 layer is of utmost importance for the mechanical properties of the single fibre [5]. During papermaking, wood fibres are processed which removes mainly lignin, but to some degree also hemicellulose and cellulose. The processing results in the removal of most of the primary wall layer as well as in a (partial) collapse of the fibre’s lumen. Furthermore, mechanical processing can cause local damage to the fibre. [10]

Since pulp fibres have lengths of a few millimetres, directly mounting them in tensile testing devices is difficult. Several adaptions to instruments have been undertaken, all with their own benefits and disadvantages [11]. In most cases the problem of mounting is solved with either custom-made sample holder systems consisting of a polymeric or metallic holder to which the fibre is attached with an epoxy glue [12–15] or micro-robotic grips [16].

Most polymers and biological materials exhibit not only elastic but also viscous characteristics, meaning that there is a time-dependent relationship between stress and strain [17]. It is well-known that paper as well as its individual fibres exhibit viscoelastic behaviour that is further influenced by temperature and moisture [11, 18–20].

Pulp fibres exhibit a complex structure; however, its main constituent cellulose is a biopolymer. For polymers, a main cause for viscoelastic behaviour is molecular rearrangement of the macromolecules. By applying stress to a viscoelastic material, parts of the polymer chain are repositioned in response to the stress, which causes creep of the material. During the repositioning, a back stress is created, which causes the material to return to its original form when the stress is removed [21]. Usually, polymeric materials are not perfectly ordered and exhibit amorphous as well as crystalline regions. In pulp fibres, cellulose has the highest degree of crystallinity and is the stiffest component, whereas hemicellulose and lignin are rather soft and amorphous polymers [22].

Deforming a viscoelastic material with a slow rate, results in a lower stiffness. On the other hand, if the deformation rate is applied fast, the material reacts stiffer. The material exhibits an effective stiffness which is influenced by the deformation rate during the test.

The effect of loading rate has been previously investigated for single fibres [3]. Increasing the loading rate from 0.005 Ns^−1^ to 0.5 Ns^−1^, an increase of the modulus by 20% was reported. Furthermore, the influence of constant and cyclic humidity conditions on creep behaviour has been studied for paper and pulp fibres [4, 23]. Here, one focus has been kept on hemicellulose as one of the constituents of the fibre. It was proposed that it affects the creep behaviour. This was also reported for wood [24]. The viscoelastic behaviour was found to be more pronounced for natural wood samples compared to wood samples from which the hemicellulose was removed.

Studying viscoelasticity of pulp fibres is interesting because their viscoelastic behaviour influences the properties of the final paper products—and therefore, the product’s performance. Here, the literature is lacking and this interplay between single fibre and paper properties needs further investigations.

In this work, a commercial dynamic mechanical analysis device was used to perform displacement-controlled tensile tests at different rates on single pulp and viscose fibres. Three different types of pulp fibres were chosen—chemi-thermomechanical softwood pulp (CTMP), chemical softwood pulp (CP), and unbleached softwood kraft pulp (UKP)—and one type of viscose fibre (VIS). Compared to the pulp fibres, VIS fibres consist only of cellulose, are more homogeneous across their length and exhibit less structural complexity than pulp fibres. Therefore, viscose is a good reference material for cellulosic fibres. Another reason to investigate viscose fibres is that they are spun as a continuous thread. So, they can be cut to arbitrary length. The correlation between the strain rate and the mechanical response was investigated by applying ten different strain rates ranging from 0.113% s^−1^ to 800% s^−1^—covering four orders of magnitude. For each fibre, the cross-sectional area was determined, and its span length was measured. This way, the force and displacement from the experiments were transformed into stress and strain data, respectively, and the rate-dependent modulus $E_{r}$ of each fibre at each strain rate was calculated by linearly fitting the stress–strain curve. Finally, the rate dependence of the elastic modulus was quantified.

## Materials and methods

### Sample preparation

**Figure 1:**
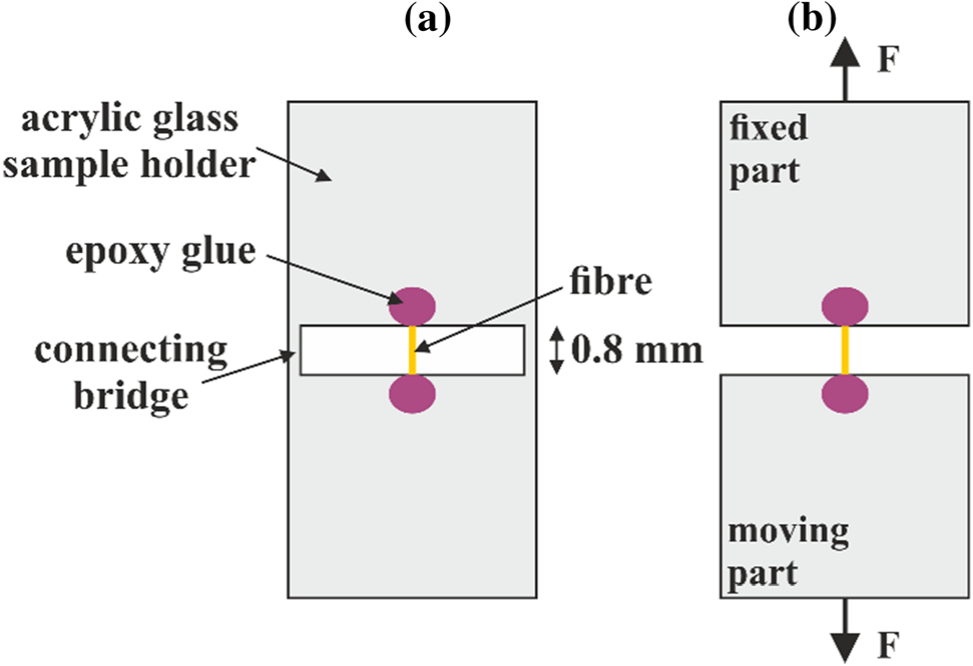
Acrylic glass sample holder with glued single pulp fibre (**a**) before and (**b**) after removal of the connecting bridges

Four types of cellulosic fibre were investigated: chemi-thermo-mechanical softwood pulp (CTMP), with a kappa number (indicating the residual lignin content) *κ* = 135, fully bleached chemical softwood pulp (CP) with *κ* = 0.8, unbleached softwood kraft pulp (UKP) with *κ* = 42, and viscose fibres (VIS). The fibres were immersed in deionised water and left to swell for 24 h. After that, droplets of the fibre suspension were deposited onto a Teflon plate. Next, the sample was covered with a second Teflon plate and dried in a pressurised rapid dryer for 1 h at 95 °C. Single fibres were identified and extracted underneath an optical microscope and glued across a 0.8 mm gap on an acrylic glass sample holder (Fig. 1a) with a two-component epoxy resin (Uhu Plus Endfest) [14, 25]. Then, a small piece of paper was pressed on each glue spot and after covering the sample with Teflon plates, everything was left for drying for two hours underneath a weight. The finished samples were left for five days in a conditioned environment (temperature *T* = 25 °C, relative humidity = 50%) before testing at the same conditions.

**Figure 2:**
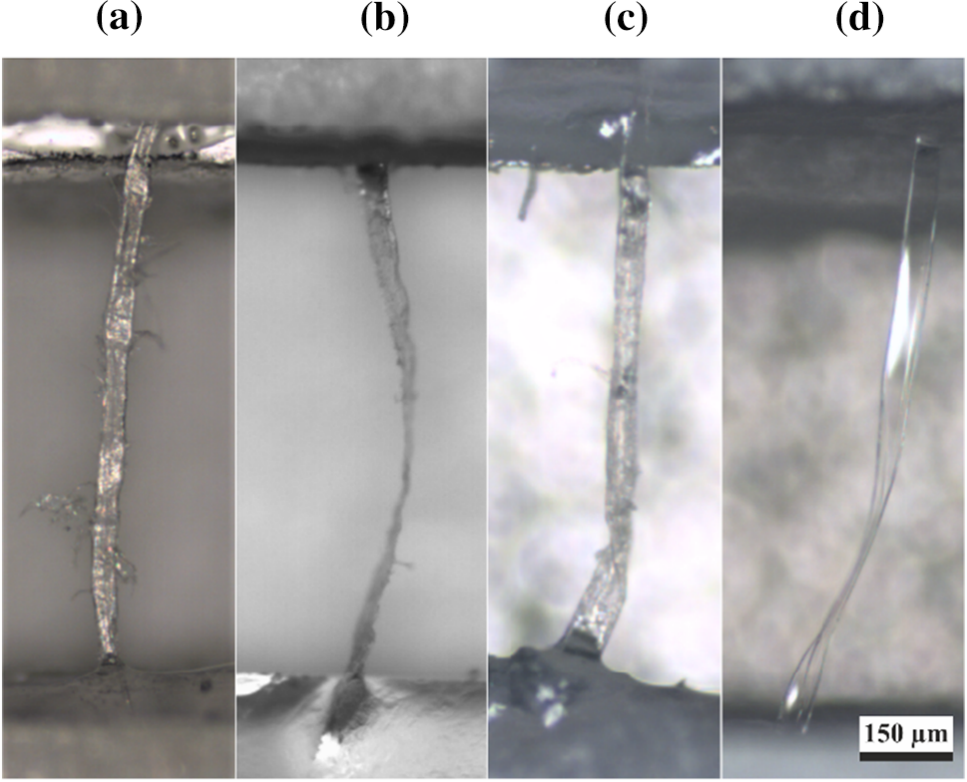
Optical images with a tenfold magnification of representative **a** CTMP, **b** CP, **c** UKP, and **d** VIS fibres. The scale bar is 150 µm

Before performing the tensile tests, the initial lengths of the prepared samples were determined by recording images with an optical microscope—as illustrated in Fig. 2—and measuring the straight-line distance between the two gluing spots.

### Dynamic mechanical analysis (DMA)

**Figure 3:**
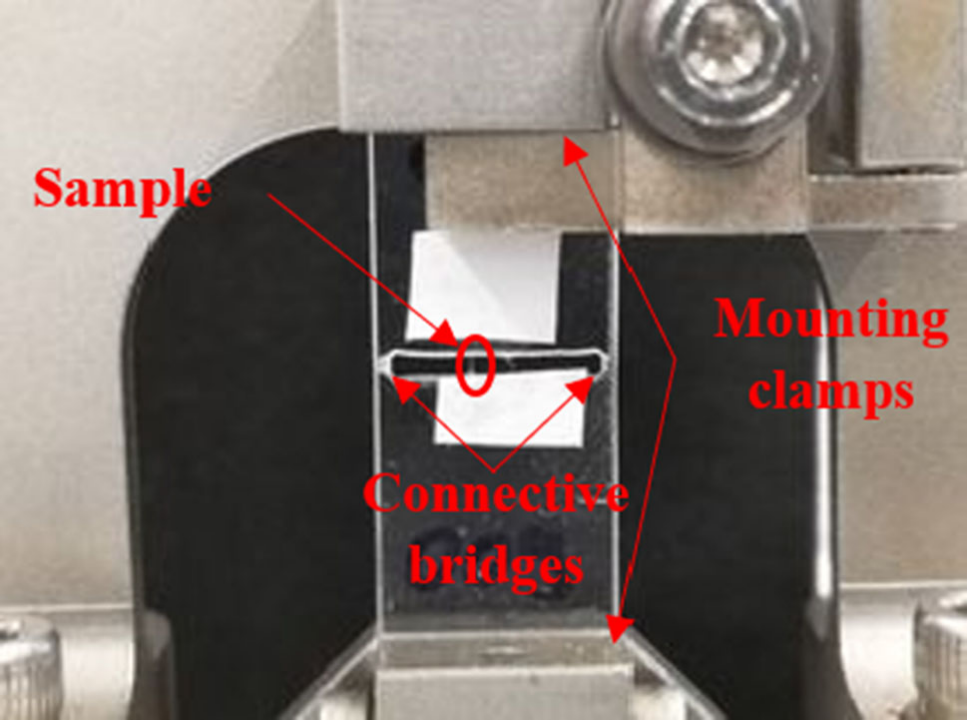
An individual fibre sample mounted vertically in the DMA device and fixated by the instrument’s clamps. The acrylic glass sample holder is still connected at the sides

The sample was mounted vertically into the DMA device (DMA850, TA Instruments, USA) as presented in Fig. 3 and after fixation of the mounting clamps, the connecting bridges were molten using a soldering device with a narrow tip.

**Figure 4:**
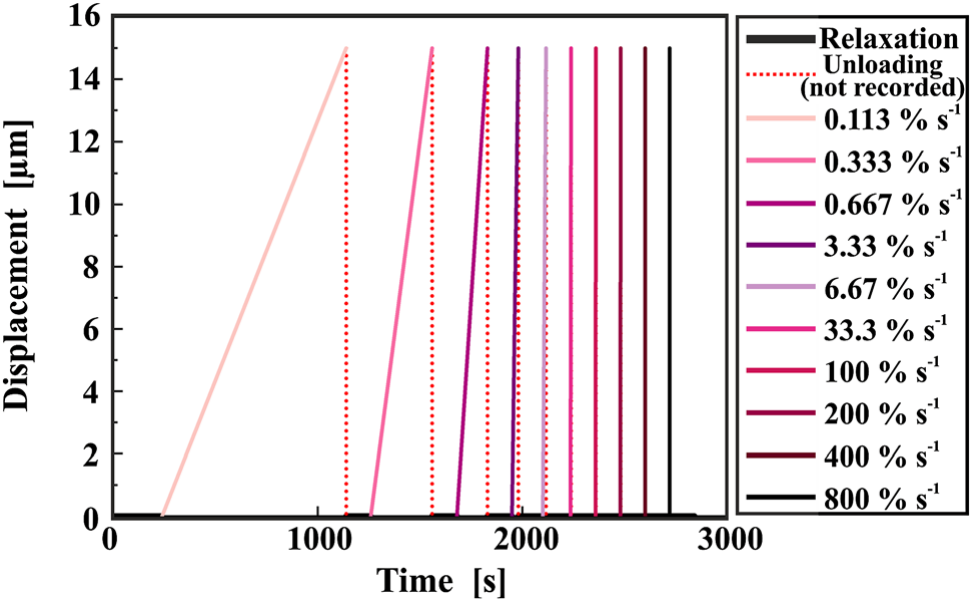
Measurement protocol for a single fibre. Ten different displacement rates were applied with a two-minute relaxation time in between each ramp. The maximum displacement was kept constant at 15 µm for each cycle. In the legend, the corresponding strain rates are indicated

The measurement consisted of a displacement-controlled cyclic protocol which is detailed in Fig. 4. In every cycle, the displacement rate was increased with a two-minute relaxation time after each ramp. In total, the experimental time for ten displacement rate cycles of a single fibre was approximately 45 min. Since the maximum displacement was kept constant at 15 µm, the applied strain rates ranged from 0.113% s^−1^ to 800% s^−1^, see Table 1.

**Table 1:** Applied displacement rates in µm s^−1^ and corresponding strain rates $r$ in % s^−1^ for every measurement cycle

Cycle No.	Applied displacement rate [µm s^−1^]	Strain rate *r* [% s^−1^]	Cycle No.	Applied displacement rate [µm s^−1^]	Strain rate *r* [% s^−1^]
1	0.017	0.113	6	5	33.3
2	0.05	0.333	7	15	100
3	0.1	0.667	8	30	200
4	0.5	3.33	9	60	400
5	1	6.67	10	120	800

To investigate the influence of loading history on the material the reverse displacement protocol was also tested for all fibre types. In the reverse protocol the first loading/unloading cycle was applied at the highest rate of 800% s^−1^, the next cycle at 400% s^−1^, and so on until the lowest rate of 0.113% s^−1^ was reached.

For both measurement protocols, the maximum displacement in every cycle was set to 15 µm, to stay in the elastic regime of the displacement curve and to ensure the fibre does not break during the measurement. Before starting the measurement, a small preload force of 10 mN (CTMP) was applied. For the more delicate CP, UKP, and VIS fibres the preload was set to 5 mN. Due to experimental limitations only the loading part of the curves was recorded. The mean initial length *L*_0_, maximum strain ε and cross-sectional area *A* for each fibre type are presented in Table 2.

**Table 2:** The mean values (± standard deviation) of the initial length $L_{0}$, maximum strain $\varepsilon$ as well as cross-sectional area $A$ for each fibre type

Fibre types (# of samples)	*L*_0_ [mm]	*ε* [%]	*A* [µm^2^]
CTMP (16)	0.80 ± 0.11	1.88 ± 0.26	440 ± 150
CP (23)	0.89 ± 0.10	1.80 ± 0.20	300 ± 90
UKP (8)	0.92 ± 0.19	1.63 ± 0.34	370 ± 120
VIS (11)	0.83 ± 0.18	1.81 ± 0.39	180 ± 15

### Cross-sectional analysis with microtome cutting

**Figure 5:**
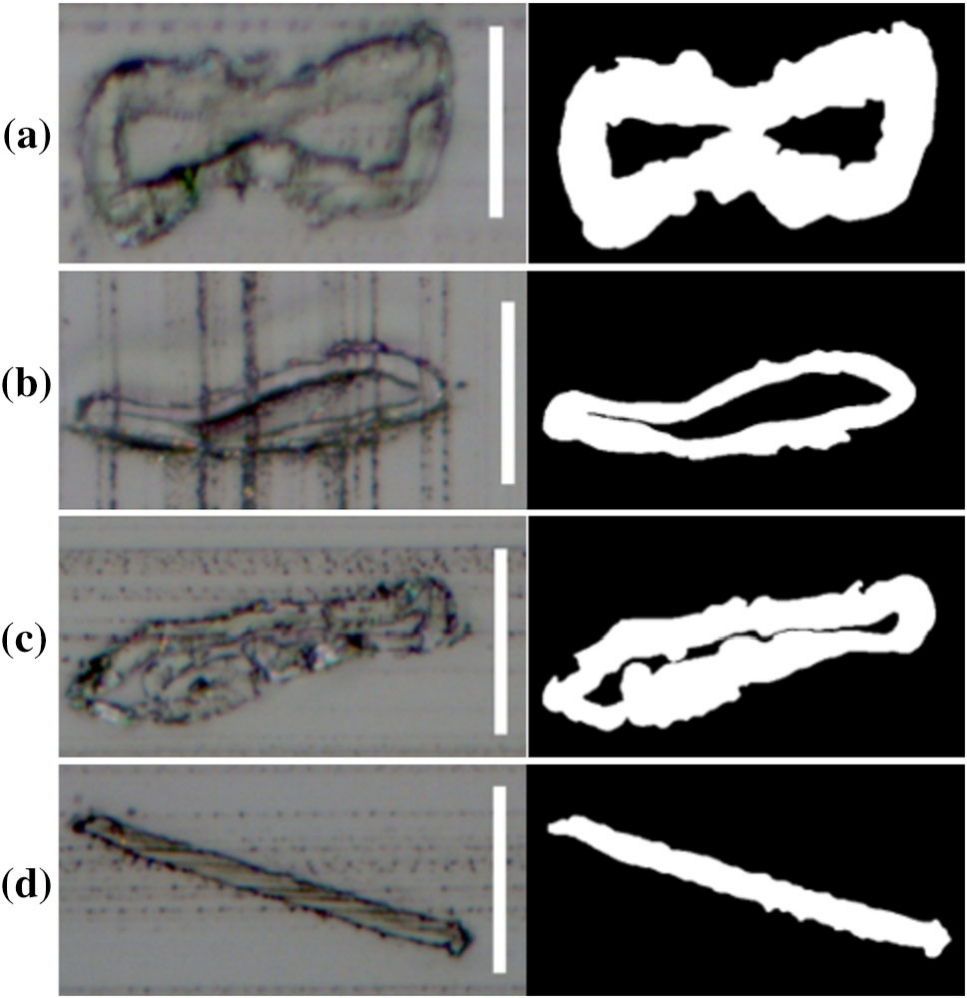
Optical (left) and binarized (right) image of **a** CTMP, **b** CP, **c** UKP, and **d** VIS. The white vertical scale bar in the optical image represents 25 µm

After the DMA measurements, the cross section of each fibre was measured. Each fibre was embedded in a glycol methacrylate resin and the resin was left 24 h to cure. The embedded sample was placed in a microtome and cut at a minimum of four different positions along the length of the fibre. For each of those cuts, an optical image of the cross section was recorded. The outlines of the cross-sectional area of the fibre were traced manually in an image analysis software. Then, the image was binarized as shown in Fig. 5 for representative examples of each fibre type [26]. The pulp fibres were characterized by a (partially) collapsed lumen (Fig. 5a–c) whereas the viscose fibre samples exhibited a uniformly flat, rectangular cross-sectional area (Fig. 5d).

The cross-sectional area of the fibre was calculated on the binarized image, as shown on the right-hand side of Fig. 5. The mean cross-sectional area for each fibre type obtained as an average from all cuts of the fibre type is also presented in Table 2.

### Data analysis

The raw data of time $t$, force $F$, and displacement $\Delta L$ were acquired from the DMA software (Trios, TA Instruments). Apart from the fibre’s deformation, possible deformations of the acrylic glass sample holder as well as the gluing spots at the fibre’s ends need to be considered. Therefore, to eliminate the compliance of the system, the displacement was corrected. This correction factor was obtained from measurement of Platinum (Pt) bands, similar to [27]. By measuring Pt bands directly mounted into the clamping of the DMA as well as glued on the sample holder (under the identical conditions as the fibres), it was possible to obtain a correction factor which was then used to correct the response of all fibres glued onto the sample holder. The correction factor values for each strain rate are listed in Table I in the Electronic Supplementary Information (ESI).

Stress and strain were determined from the force and corrected displacement data by consideration of the cross-sectional area and the initial length of each fibre. The stress was calculated by using Eq. 1:

1 $$\sigma = \frac{F}{A}$$

Where $\sigma$ is the stress [Nm^−2^], $F$ is the force [N], and $A$ is the cross-sectional area [m^2^].

The strain was obtained with Eq. 2:

2 $$\varepsilon = \frac{{\Delta L_{{{\text{corr}}}} }}{{L_{{0}} }}$$

Where $\varepsilon$ is the strain [/], $\Delta L_{{{\text{corr}}}}$ is the corrected displacement [m], and $L_{0}$ is the initial length of the fibre [m].

For each fibre and rate, the stress–strain curve was plotted, the curves were smoothed with a Savitzky–Golay filter to remove signal noise, and the rate-dependent longitudinal modulus $E_{{\text{r}}}$ was determined by linearly fitting the curve according to

3 $$\sigma = E_{{\text{r}}} \varepsilon$$

Grubbs Outlier tests were performed for each fibre type, assuming normally distributed data. The critical values were determined with the statistical significance $\alpha = 0.05$ and the number of samples $N$. In this work, the results for $E_{{\text{r}}}$ are presented as mean values with error bars representing the standard deviations.

## Results

### Cyclic testing to determine plasticity effects

The first set of cyclic testing experiments was aimed at controlling and eliminating plasticity effects on the measurement of the elastic modulus. In these experiments, chemi-thermomechanical pulp (CTMP) and chemical pulp (CP) fibres were tested, and the force rate was kept constant (loading and unloading rate: 10 mN s^−1^), but the force level was increased after each loading–unloading cycle. Starting with a preload according to the values in this work, the fibre was first loaded to 15 mN and after unloading, a 90 s relaxation time was included until the next force ramp to a 5 mN higher force was started. This continued until the fibre broke.

**Figure 6:**
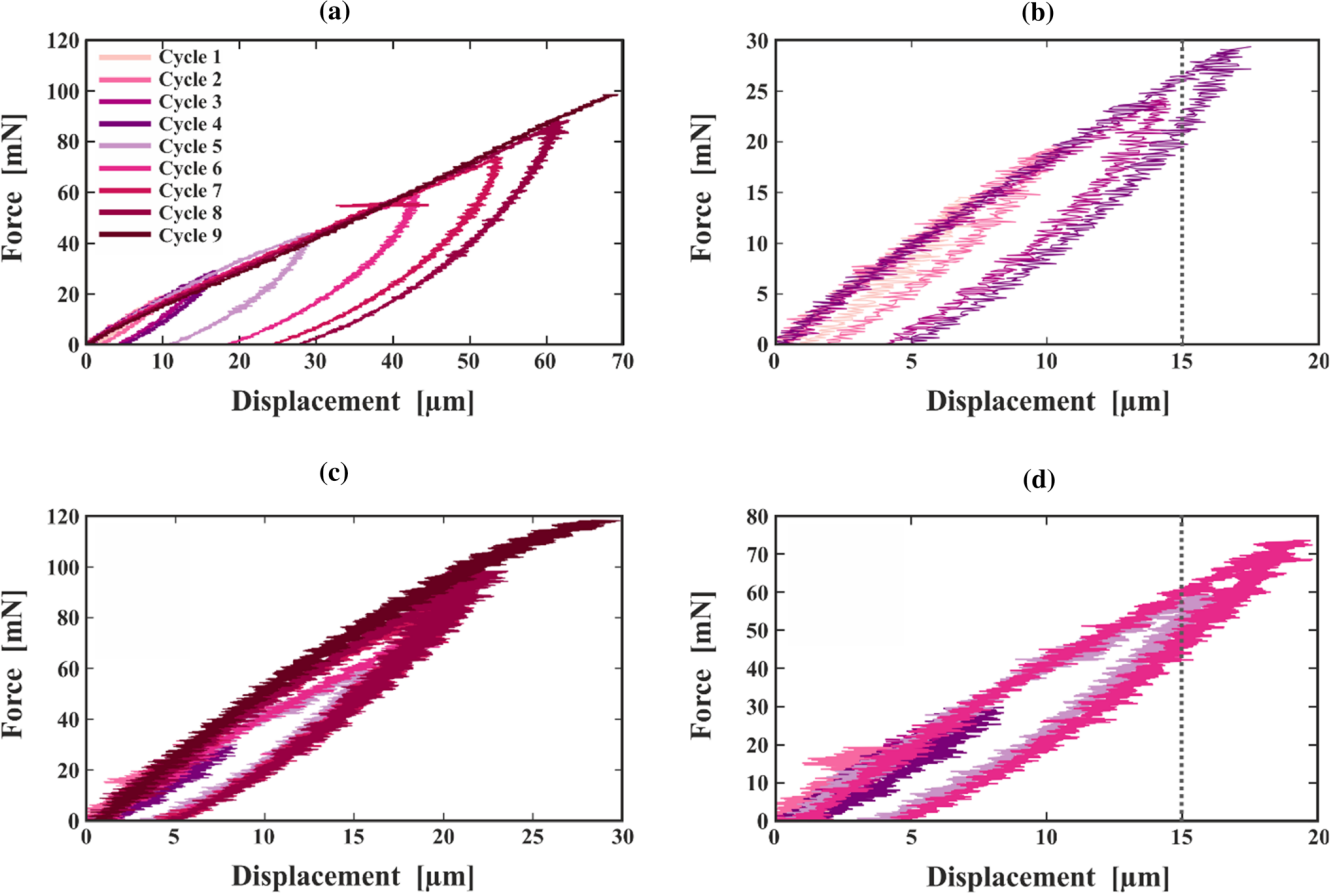
Representative force–displacement curves from force-controlled tensile experiments until breakage of (**a**, **b**) CTMP and (**c**, **d**) CP fibres. In (**b**) a zoom-in into the first 20 µm of displacement of (**a**) are provided displaying cycle 1–4. The same is plotted in (**d**) for (**c**) displaying cycle 1–6. The dashed line indicates 15 µm displacement

In Fig. 6, the resulting force–displacement curves are presented for CTMP and CP. As can be seen in Fig. 6a, the CTMP fibre breaks after eight cycles of loading and unloading in the loading phase at a force of about 100 mN. It is observable that the slope of the loading curve decreases at higher force levels. In Fig. 6b, a zoom-in is presented, illustrating that it takes four cycles to a maximum force of 30 mN until the displacement surpasses 15 µm. At forces greater than 20 mN a permanent plastic deformation is observed which is clearly distinguishable from the signal noise. The CP fibre is illustrated in Fig. 6c with a zoom-in in Fig. 6d. Here, the higher noise level is due to the lower maximum displacement (30 µm compared to 70 µm for CTMP). The breaking force for CP fibres is slightly higher than for CTMP at about 120 mN. Due to the signal noise, it is difficult to interpret the slope of the curves. In the zoomed displacement region in Fig. 6d, six cycles of loading and unloading with a maximum force of 70 mN are needed to cross the 15 µm displacement limit. Similar to CTMP, also here a permanent set is observed for forces higher than 50 mN.

From the results obtained in these force-controlled measurements, we concluded that using a 15 µm maximum displacement in a continuous measurement protocol for single fibres is sufficiently small to avoid plastic deformation while providing a reasonable signal-to-noise ratio.

### Evaluation of stress–strain curves at different strain rates

**Figure 7:**
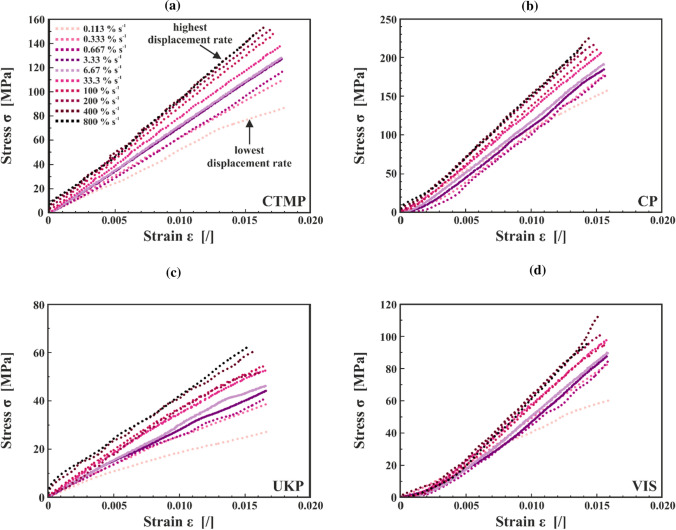
Stress–strain curves of all the strain rates for representative samples of (**a**) CTMP, (**b**) CP, (**c**) UKP, and (**d**) VIS. The light-pink dotted line represents the lowest strain rate (0.113% s^−1^) and the black dotted line the highest strain rate (800% s^−1^). Please note that the distance between the dots is not equal for all curves due to different sampling rates and that the curves were smoothed to remove signal noise

In Fig. 7, the stress–strain curves for each fibre type at all strain rates are presented for a representative sample. For each fibre type, between eight and 23 samples were tested. Ten different strain rates ranging from 0.113% s^−1^ to 800% s^−1^ were performed for each sample with a maximum displacement of 15 µm. The curves have been offset corrected. The stress–strain curves without offset correction are presented in Figure I in the ESI. For all fibre types, the lowest strain rate (0.113% s^−1^) shows a deviating response. Especially at higher strain values it levels off more than the curves at other strain rates. It seems that in the first cycle a minor permanent deformation leads to a slight stiffening of the sample. By turning the experimental protocol around and starting it with the highest strain rate, a similar behaviour is found. Here, the highest strain rate is the first cycle and shows a deviating response (please refer to Figure II in the ESI).

Comparing the three pulp fibre types in Fig. 7a–c, CP exhibits the highest stress values followed by CTMP and UKP. UKP has maximum stresses which are about a factor 2–3 lower compared to the other two pulp types. The shape of the curves also shows some difference. Whereas CTMP and UKP are quite linear, CP and VIS (Fig. 7d) have a lower initial slope which is increasing with higher strain values. However, there is roughly a factor two between the maximum stresses of CP and VIS. For this work, individual VIS fibres were also tested without the sample holder (VIS no SH). These fibres were clamped directly into the DMA with a span length of 4 mm. Testing them according to the described protocol, the maximum strain for these samples was about 0.38% which is a factor of about five lower compared to the maximum strains for the samples within the sample holder (see Table 2). The VIS no SH samples do not show any significant deviation in behaviour in their stress–strain curves, therefore, the results are not included in Fig. 7, but later the modulus and slope results will be presented as a comparison to the sample holder samples. In Figure III in the ESI, information on the measurements of VIS no SH is summarized.

**Figure 8:**
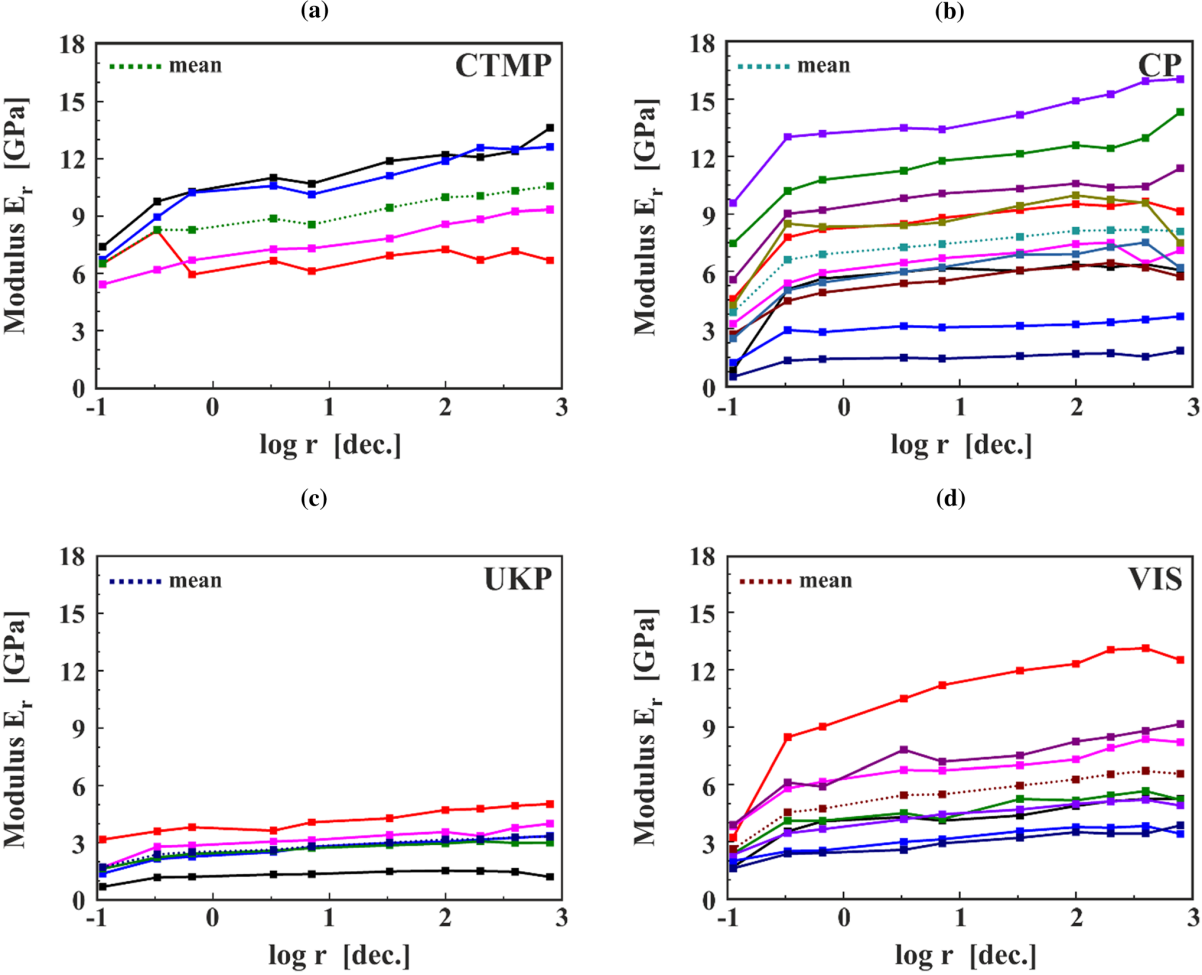
The change of the modulus in dependence of the strain rate r for samples (**a**) CTMP, (**b**) CP, (**c**) UKP, and (**d**) VIS. The squares indicate measurements whereas the lines are just a guideline to the eye. Each colour represents a different fibre sample. The mean value for each fibre type is illustrated with squares connected by dotted lines

After calculation, the moduli for each rate and each sample are presented in Fig. 8, where the abscissa is logarithmic. The modulus $E_{{\text{r}}}$ clearly displays a dependence on the value of $r$ for all fibre types. Due to the difficulties in preparation and handling of the samples, there is a different number of successfully measured samples: four samples of CTMP (Fig. 8a), eleven samples of CP (Fig. 8b), five samples of UKP (Fig. 8c), and eight samples of VIS (Fig. 8d). Although there is a large scatter even between samples of the same fibre type, a trend of linear increase of the tensile stiffness can be observed for all five types of fibres and this is further confirmed when observing the mean values of modulus $E_{{\text{r}}}$, represented by the dotted-lined curves. Furthermore, a large increase in $E_{{\text{r}}}$ can be observed between the first and the second cycle measurements and a slightly smaller change, either as an increase or a decrease between the ninth and tenth cycle measurement. Both phenomena influence the overall slope of each curve.

**Figure 9:**
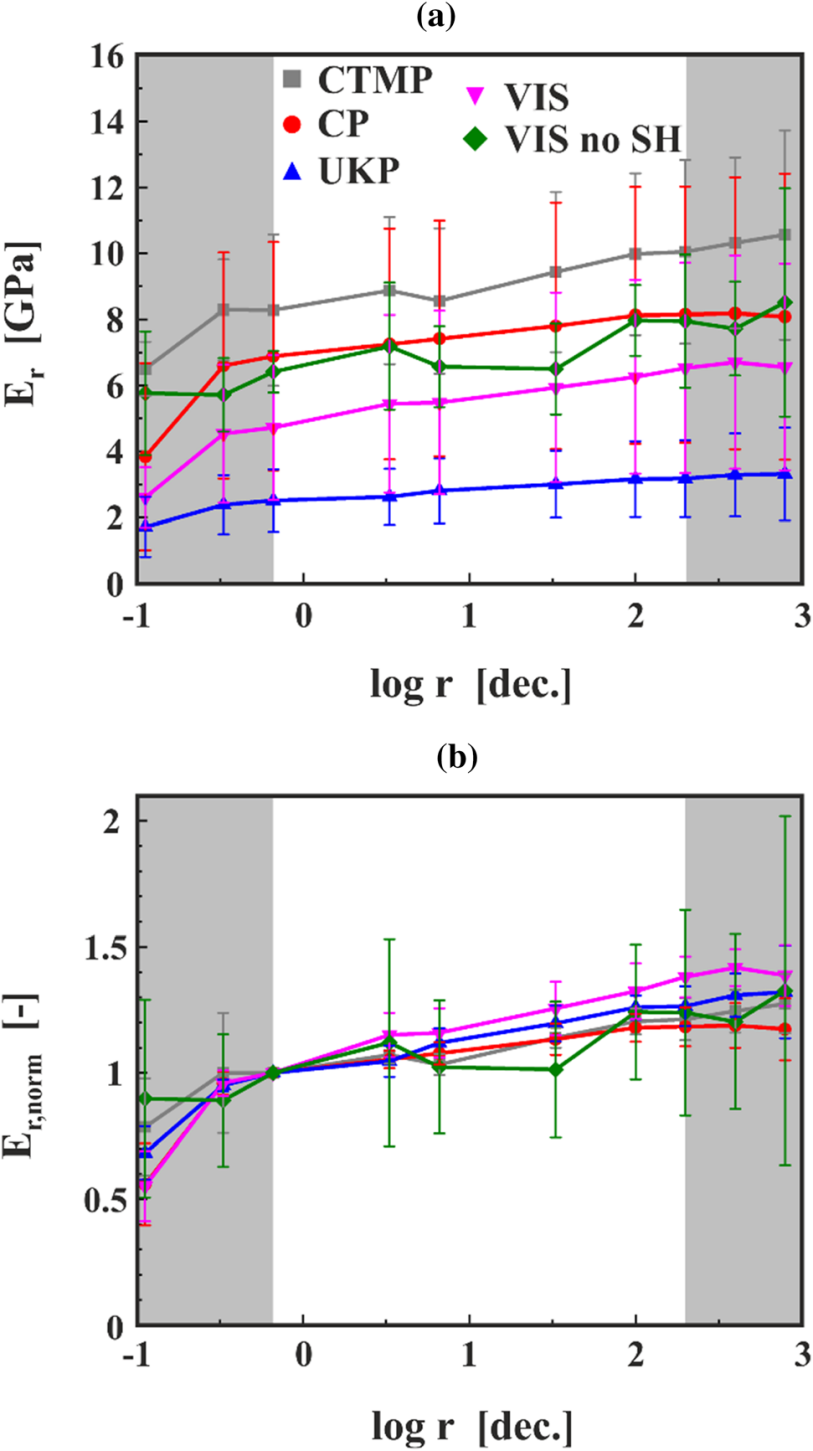
The change of the (**a**) mean moduli $E_{{\text{r}}}$ and the (**b**) normalized moduli $E_{{\text{r,norm}}}$ in dependence of the logarithmic strain rate $r$ for CTMP, CP, UKP, VIS, and VIS no SH fibres.

In Fig. 9, the mean values of $E_{{\text{r}}}$ are plotted against the logarithm of the strain rate $r$ for each type of fibre with the error bars representing standard deviations. Due to the discrepancies at the lowest and highest rates and their strong influence on the slope, the results from the first two and the last two cycles were not used to determine the correlation between $E_{{\text{r}}}$ and log $r$. This is indicated in Fig. 9 with the grey areas on the left and right side of the plot. By comparing the change of the mean modulus in dependence of the logarithmic strain rate (Fig. 9a), a linear increase of $E_{{\text{r}}}$ can be observed with the logarithm of the strain rate $r$. CTMP displays the highest value of $E_{{\text{r}}}$, followed by CP, VIS no SH, VIS and UKP. Here, a large scattering is visible for all pulp fibre types, only UKP is clearly showing lower $E_{{\text{r}}}$ values than CTMP and CP. When the modulus values are normalized by dividing them with the $E_{{\text{r}}}$ from the third cycle measurement, which is also the first value used to calculate the slope, a unitless and material specific modulus $E_{{\text{r,norm}}}$ is determined (Fig. 9b). By plotting $E_{{\text{r,norm}}}$, the relative changes of the $E_{{\text{r}}}$ in dependence of the $r$ can be observed. One can clearly see that there is only a small difference in the loading rate dependency of $E_{{\text{r}}}$ for the different types of fibres.

As mentioned earlier, the lowest strain rate shows a deviating behaviour already in the stress–strain curves. Since this tensile test took about 15 min and therefore much longer than the relaxation time between different rates (2 min), possible effects coming from that had to be addressed. Therefore, the experimental protocol was reversed, so it started with the highest strain rate and decreased to the lowest strain rate. The rest of this reverse protocol was identical to the normal one illustrated in Fig. 4. However, no major differences were observed. The first cycle (now at the highest strain rate) again showed a deviation similar to the normal protocol for all samples (Figure II in ESI). Please also note that for this protocol only a limited number of samples has been evaluated (three samples per fibre type).

### Quantification of the strain rate dependence

**Figure 10:**
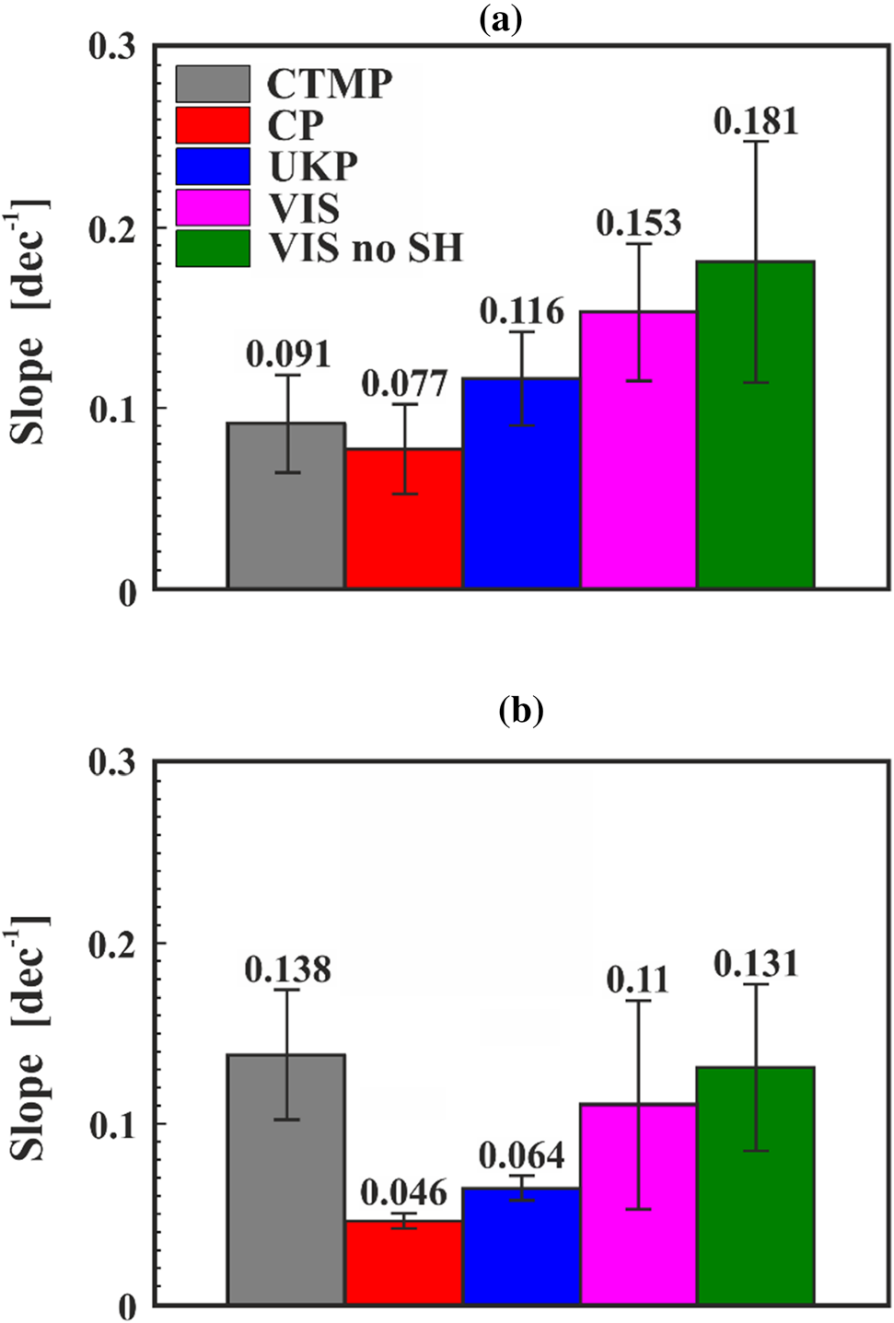
Loading rate dependency of the normalized moduli $E_{{\text{r,norm}}}$ for CTMP, CP, UKP, VIS, and VIS no SH fibres as a function of the logarithmic strain rate $r$ experimentally obtained via (**a**) the normal protocol and (**b**) the reverse protocol. The numbers at the top of the bar indicate the mean slope values for each fibre type. A value of 0.091 for CTMP for example means that for a tenfold increase in strain rate the modulus is increasing by 9.1%

To quantify the influence of the rate $r$ on the modulus $E_{{\text{r}}}$ further, the slope of the normalized moduli (as illustrated in Fig. 9b) was determined for each type of fibre. The values of these slopes in both the normal and the reverse measured protocol are presented in Fig. 10. The slope was calculated per decade (dec.^−1^) which means that for every increase of the strain rate by a factor of 10, the modulus will increase by factor of the respective slope. A value of 0.091 for CTMP for example means that for a tenfold increase in loading rate the modulus increases by 9.1%. Owing to the linear increase in modulus over log of the loading rate (cf. Figure 9) the influence of loading rate on measured E-modulus can be condensed to one single number for each fibre type. By comparing the normalized slopes of the normal (Fig. 10a) and reverse (Fig. 10b) protocol, it can be seen that all types of fibres except for CTMP follow the same trend. Excluding CTMP, CP has the lowest slope value, and the viscose fibre samples the highest. Furthermore, the slope results for the reverse protocol are nearly a factor two lower. However, it should be noted that individual fibres were only tested using one of the protocols, and not both. Hence, the observations do not form pairs that can be directly compared. Therefore, there is quite a scattering of the data and the observed difference in slope of the different protocols could be a result of the inhomogeneity of the fibres themselves. The pulp fibre types show a decrease of about 40%–50% for the mean slope between normal and reverse protocol, whereas for the viscose samples with and without sample holder the difference is about 30%.

## Discussion

All fibre samples measured in this work exhibited rate-dependent behaviour. Pulp fibres in general are quite inhomogeneous and exhibit a high degree of scattering. The variation within a fibre type is high as can be seen for CP in Fig. 8b. Even for more uniform natural fibres like bast, scattering can limit the resolution of the rate dependence of the modulus [28]. Furthermore, handling of the single fibres is not straightforward which leads to lengthy sample preparation with some sample reject also in the initial starting period of the experiments. Therefore, only a limited number of specimens can be tested in a reasonable time frame.

With the performance of loading–unloading experiments at different force levels at the start of this study, possible plastic effects during the experiments can be excluded. As visible in Fig. 6, for the chosen maximum displacement of 15 µm the fibres have low plastic deformation.

By reversing the experimental protocol, it was possible to exclude relaxation effects which could have been caused by the very slow first rate and insufficient relaxation time afterwards. The first displacement cycle always exhibits some deviation and most likely some irreversible deformation. Pulp fibres frequently contain locally damaged zones after processing, such as micro-compressions. This first cycle might be straightening these zones [29].

The rate-dependent moduli for all fibre samples show an increasing value with increasing rate, Fig. 9. Looking at the slope values in Fig. 10, CP always has the lowest values. Bleaching removes the amorphous lignin and hemicellulose from the pulp fibres. Due to the removal of these materials, the overall degree of crystallinity is higher in pulp fibres after bleaching since mostly cellulose is left [22]. In the literature, it was found that wood containing hemicellulose exhibit a stronger creep behaviour compared to wood which had the hemicellulose removed [24]. For fibres, this was also investigated, but the results are not as clear [23]. The viscose fibre samples (VIS and VIS no SH)—consisting only of cellulose—show the highest slope values. Viscose fibres are industrially produced in a spinning process and exhibit a different cellulose crystal structure with a lower degree of crystallinity compared to pulp fibres [30]. Due to the draw in the production process after the spinning they consistently have a high degree of longitudinal fibril orientation [30]. For VIS no SH, only three samples were measured, and the span length of these samples was much longer than that of the fibres glued into the sample holder. This resulted in smaller strains for the VIS no SH fibres during the experiments (0.4% strain compared to 1.8% for VIS fibres). Due to these facts, it can be assumed that there is no significant difference between VIS and VIS no SH. From another perspective, one can also see this similarity in behaviour as a proof that there is no significant influence of viscoelastic or plastic properties of the sample holder system, which also consists of polymeric materials, on the measured data and that the correction of the data works.

The relation between the three pulp fibre types is difficult to interpret due to the opposite trend for CTMP with the two different protocols. The two mean values for the slope are still in the margin of each other’s standard deviation, so the difference in slope values might be just an effect from the natural variability. CTMP fibres are quite similar to wood fibres. The chemical composition is not strongly altered, but the fibres have been mechanically processed and the lumen is (partially) collapsed. This could be the cause for the high scattering and, eventually, not enough fibres were measured to obtain stable values.

The UKP fibres exhibit—compared to CTMP and CP—a very low absolute value for the modulus as presented in Fig. 8c. These fibres have been subject to investigations before and there their mean longitudinal modulus was four times higher, though they exhibited considerable scattering [31]. Still, these fibres also exhibit rate-dependent behaviour. The results for the slope values of the normal and reverse protocol show a significant difference of the values, roughly a factor of 2. It can be assumed that also here not enough data has been compiled. Due to the small modulus values, maybe some experimental complications happened, which have not been recognized, also the stress–strain curves in Fig. 7c exhibit rather low stress values compared to the other fibres. Despite this discrepancy, the authors decided to present the UKP data since the results for the normalized modulus show a reasonable trend. Furthermore, this UKP data is a good example to emphasize the difficulties to obtain straightforward experimental data from single pulp fibres.

## Conclusions

In this work, different types of cellulose fibres–industrial wood pulp (CTMP, CP, UKP) fibres and viscose (VIS, VIS no SH) fibres–were shown to display viscoelastic behaviour, which was investigated via the relationship between the longitudinal modulus of the material and the applied strain rate by tensile testing. For each fibre sample, ten strain rates ranging from 0.113% s^−1^ to 800% s^−1^ were applied. The rate-dependent modulus $E_{{\text{r}}}$ of each fibre at each strain rate was calculated by linearly fitting the stress–strain curves.

With the determination of the slopes for each fibre type in both measurement protocols, an influence of the strain rate on the modulus was found, proving that the strain rate has a significant influence on the results of the tensile testing of cellulosic fibres. For each tenfold increase in strain rate we found an increase in longitudinal modulus between 5 and 7% (bleached chemical pulp CP) and 11–18% (viscose VIS), see Fig. 10. In the literature, similar results were reported [3]. Here, the modulus of single fibres increased by 20% with increasing loading rate from 0.005 Ns^−1^ to 0.5 Ns^−1^.

To exclude possible plastic and relaxation effects, two different experimental approaches have been complementing this work. Since pulp fibres exhibits a high variability, it is tricky to find a procedure to fully eliminate plasticity and relaxation, but in this work the effort was made to avoid it as far as possible. First, loading–unloading experiments in a force-controlled schedule were performed to analyse the material behaviour until breakage. Very little plasticity was observed, and it was possible to select a maximum displacement of 15 µm which is a compromise between possible deformation and signal-to-noise ratio of the measurement. At the beginning of the rate-dependence experiments, the lowest strain rate was performed with a duration of 15 min which was longer than the relaxation time afterwards. Therefore, a reverse protocol was established, going from highest to lowest rate, to eliminate any influence from relaxation processes.

Except for one fibre type (CTMP), both protocols exhibit the same trend. VIS fibres exhibit the highest slope values, and therefore, the highest effect of rate, whereas CP fibres have the lowest. Due to a lack of reliable statistics, there is some difficulty in the interpretation of CTMP and UKP fibres. Furthermore, the mean values between both protocols show some difference, however, the standard deviation is still high which indicates that more measurements are needed. In future works, it might be favourable to test the same fibre with both protocols to eliminate the effect from sample variation.

In summary, it was presented that there is a significant rate dependence of the modulus for all cellulosic fibre types. For fibre tensile testing this means that the loading/displacement rate must be carefully controlled. For mechanical modelling of cellulosic fibres an increase in longitudinal elastic modulus for increasing rates needs to be considered. For each tenfold increase in loading rate we found an increase in the modulus up to nearly 20%—depending on the fibre type.

## Supplementary Information

Below is the link to the electronic supplementary material.

Supplementary file1 (PDF 311 kb)

## Declarations

### Data availability

All the data is available under: 10.3217/6qtya-fmf85.
