# Supplementary material for: The effect of the strain rate on the longitudinal modulus of cellulosic fibres
Source: J Mater Sci. 2022 Sep 22;57(36):17517–29. doi: 10.1007/s10853-022-07722-7 (PMC9529687; doi:10.1007/s10853-022-07722-7)
Supplement: Supplementary file 1 — Supplementary file1 (PDF 311 kb) [file 10853_2022_7722_MOESM1_ESM.pdf]

Table I: Correction factor values obtained for each strain rate with a single Platinum (Pt) wire and a Pt-wire fixed in a sample holder (Pt-S).

**Applied displacement rate [µm s^{-1}]**	**Strain rate [% s^{-1}]**	**Pt [mm/mN]**	**Pt-S [mm/mN]**	**Correction factor Pt - Pt-S [mm/mN]**
**0.017**	**0.113**	9.27E-05	4.21E-06	8.85E-05
**0.05**	**0.333**	9.43E-05	3.92E-05	5.51E-05
**0.1**	**0.667**	9.13E-05	3.83E-05	5.30E-05
**0.5**	**3.33**	8.76E-05	3.65E-05	5.12E-05
**1**	**6.67**	9.25E-05	3.57E-05	5.68E-05
**5**	**33.3**	9.25E-05	3.34E-05	5.91E-05
**15**	**100**	9.22E-05	3.24E-05	5.98E-05
**30**	**200**	9.53E-05	3.20E-05	6.33E-05
**60**	**400**	9.06E-05	3.20E-05	5.86E-05
**120**	**800**	9.21E-05	3.31E-05	5.90E-05

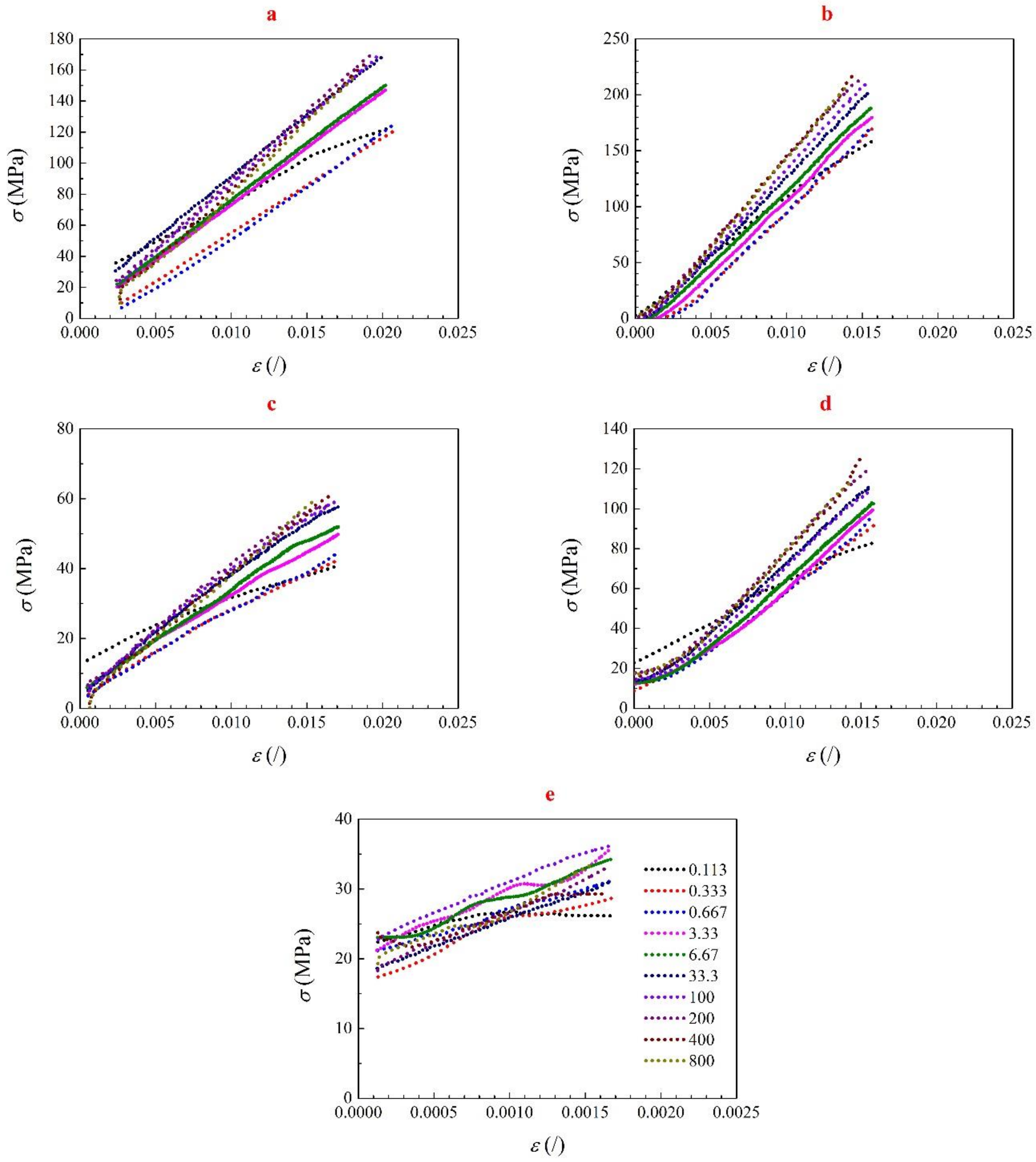

Figure I: Behaviour of the measured stress-strain curves with the change of the strain rate for representative samples of (a) CTMP, (b) CP, (c) UKP, (d) VIS and (e) VIS no SH without offset correction.

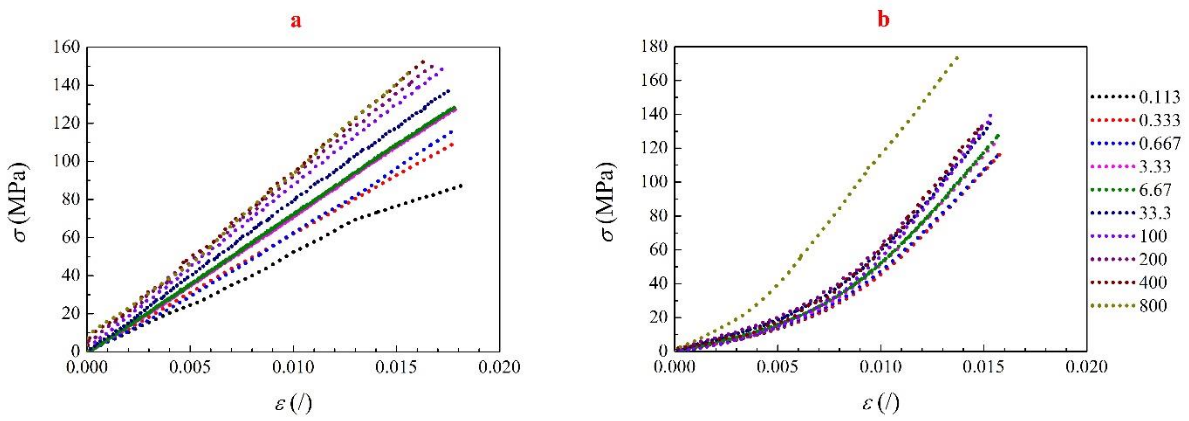

Figure II: Behaviour of the stress-strain curves with corrected offset for representative samples of CTMP measured with (a) the normal and (b) the reverse protocol.

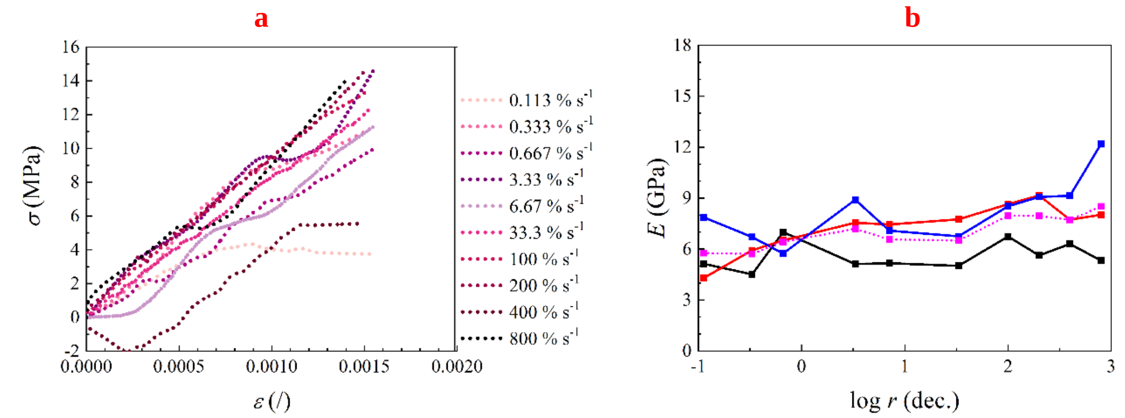

Figure III: Viscose fibres without sample holder (VIS no SH) samples: (a) Stress-strain curves with offset correction for the different strain rates, (b) change of the modulus in dependence of the logarithmic strain rate for three VIS no SH samples. The pink squares in (b) correspond to the mean value and the lines are just a guide to the eye.
